# *Patience*, *Persistence and Pragmatism*: Experiences and Lessons Learnt from the Implementation of Clinically Integrated Teaching and Learning of Evidence-Based Health Care – A Qualitative Study

**DOI:** 10.1371/journal.pone.0131121

**Published:** 2015-06-25

**Authors:** Taryn Young, Anke Rohwer, Susan van Schalkwyk, Jimmy Volmink, Mike Clarke

**Affiliations:** 1 Centre for Evidence-based Health Care, Faculty of Medicine and Health Sciences, Stellenbosch University, Cape Town, South Africa; 2 South African Cochrane Centre, South African Medical Research Council, Cape Town, South Africa; 3 Division of Community Health, Faculty of Medicine and Health Sciences, Stellenbosch University, Cape Town, South Africa; 4 Centre for Health Professions Education, Faculty of Medicine and Health Sciences, Stellenbosch University, Cape Town, South Africa; 5 All Ireland Hub for Trials Methodology Research, Queen’s University Belfast, Belfast, Northern Ireland; Canadian Agency for Drugs and Technologies in Health, CANADA

## Abstract

**Background:**

Clinically integrated teaching and learning are regarded as the best options for improving evidence-based healthcare (EBHC) knowledge, skills and attitudes. To inform implementation of such strategies, we assessed experiences and opinions on lessons learnt of those involved in such programmes.

**Methods and Findings:**

We conducted semi-structured interviews with 24 EBHC programme coordinators from around the world, selected through purposive sampling. Following data transcription, a multidisciplinary group of investigators carried out analysis and data interpretation, using thematic content analysis. Successful implementation of clinically integrated teaching and learning of EBHC takes much time. Student learning needs to start in pre-clinical years with consolidation, application and assessment following in clinical years. Learning is supported through partnerships between various types of staff including the core EBHC team, clinical lecturers and clinicians working in the clinical setting. While full integration of EBHC learning into all clinical rotations is considered necessary, this was not always achieved. Critical success factors were pragmatism and readiness to use opportunities for engagement and including EBHC learning in the curriculum; patience; and a critical mass of the right teachers who have EBHC knowledge and skills and are confident in facilitating learning. Role modelling of EBHC within the clinical setting emerged as an important facilitator. The institutional context exerts an important influence; with faculty buy-in, endorsement by institutional leaders, and an EBHC-friendly culture, together with a supportive community of practice, all acting as key enablers. The most common challenges identified were lack of teaching time within the clinical curriculum, misconceptions about EBHC, resistance of staff, lack of confidence of tutors, lack of time, and negative role modelling.

**Conclusions:**

Implementing clinically integrated EBHC curricula requires institutional support, a critical mass of the right teachers and role models in the clinical setting combined with patience, persistence and pragmatism on the part of teachers.

## Introduction

In many low and middle income countries, healthcare professionals and decision makers are often simultaneously challenged by a significant burden of infectious diseases, a rising epidemic of chronic diseases of lifestyle, and the on-going consequences of violence and injuries [1]. This creates the need for enhancing human, health systems and research capacity to address the prevention and management of multiple conditions [2], and to ensure that scarce resources are used effectively and efficiently [3, 4].

Evidence-based health care (EBHC) is an approach to delivering health care which has the potential to address these needs by fostering specific skills needed to access, appraise, interpret and apply knowledge. While widely recognised as an important competency for the health professional of the 21st century, EBHC teaching and learning, at both student and professional levels, is often haphazard, fragmented or non-existent. The focus is often on whether or not to teach EBHC, rather than on how best to teach EBHC [5, 6].

Although various teaching and learning strategies exist, EBHC remains difficult to teach [7] perhaps because in some instances the conceptualisation of the EBHC model lacks complete and clear description. Teaching of EBHC can be done as standalone sessions or be integrated within clinical practice. It may be offered using face:face contact sessions, online learning or both, and can include both individual and group teaching and collaborative learning. Furthermore, the teaching approach may use directed learning or self-directed (e.g. problem-based) learning. The content of EBHC curricula usually emphasises the five steps of EBHC (acknowledge uncertainty and phrase clear question, search for research evidence, critically appraise and interpret the evidence, consider application and evaluate) and key competencies required to practice EBHC also build on these steps [8, 9]. Findings from an overview of systematic reviews on the effects of EBHC teaching and learning approaches [10] and a recent randomised trial [11] show that clinically integrated teaching and learning strategies, with assessment, are the best options for improving EBHC knowledge, skills and attitudes. In addition, a hierarchy of EBHC teaching and learning has been described which proposes three levels of EBHC teaching and learning activities—*“Level 1*, *interactive and clinically integrated activities; Level 2(a)*, *interactive but classroom based activities; Level 2(b)*, *didactic but clinically integrated activities; and Level 3*, *didactic*, *classroom or standalone teaching*.*”* [12]

Yet, little is known about how to implement clinically integrated EBHC teaching and learning. A popular textbook on practicing and teaching EBHC [13] identifies approaches that should be foregrounded in teaching and those that should be avoided. It highlights actual learning needs, balancing active and passive learning, connecting new knowledge with what is already known and seamlessly integrating EBHC into patient care decisions (Table 1). Furthermore, it is emphasised that one needs to focus teaching and learning on real clinical decisions [14].

**Table 1:** EBHC teaching and learning tips and mistakes to avoid [13].

Teaching and learning strategies to include	Mistakes to avoid
Base teaching on real clinical decisions and actions	Emphasis on doing research instead of using research to inform decision making
Focus on learners actual learning needs	When learning how to do statistics is emphasised over how to interpret statistics
Passive and active learning used in balanced manner	Only finding fault with research
Involves everyone in the team	Promoting EBHC instead of clinical expertise
What is already known connected with new knowledge	Focus on critical appraisal only
Teacher explicit about appraisal of evidence	Disconnect from clinical process and team’s learning needs
EBHC seamlessly integrated into patient care decision	Amount of teaching exceeds available time and learners’ attention
Provides foundation and tools for lifelong learning	Not including time to learn in between formal sessions

As part of a process to enhance EBHC teaching and learning at an academic institution in South Africa, we assessed lessons learnt from those who have successfully implemented, or who have attempted and failed to implement, clinically integrated EBHC teaching and learning locally and in other parts of the world. The study objectives were to describe approaches used to clinically integrate EBHC teaching and learning in medicine and health sciences programmes, and to determine barriers and facilitators in the teaching and learning of EBHC in an integrated manner.

## Methods

Our study was situated within an interpretivist paradigm which sought to understand specific phenomena, recognising that meaning is constructed during the research process and as a result of engagement between researcher and participants [15]. This was a qualitative study based on the perceptions of key informants generated during a series of semi-structured interviews. We used purposive sampling to select key informants with experience in implementing clinically integrated teaching and learning of EBHC involving health professions students studying for their first degree (Table 2). The Faculty of Medicine and Health Sciences, Stellenbosch University, Health Research Ethics Committee provided ethics approval for the study (S12/10/262b). The consolidated criteria for reporting qualitative research (COREQ) [16] (S1 file) and the standards for reporting qualitative research [17] guided the reporting of the study.

**Table 2:** Key definitions.

*Clinically integrated teaching and learning*	Teaching and learning of EBHC integrated in clinical practice, whether interactive or didactic, compared to classroom based teaching. [12]
*Academic programmes*	A higher education programme for any healthcare professional.
*Medicine or health sciences student*	A college or university student who has not yet received a health professions degree (this included both undergraduate and graduate medical programmes) and excluded postgraduate students.
*Evidence-based health care*	Evidence-based medicine (EBM) was first defined by Gordon Guyatt as “*an ability to assess the validity and importance of evidence before applying it to day-to-day clinical problems”* [9]. David Sackett (1996) then furthered this definition as “*the conscientious*, *explicit and judicious use of the current best evidence in making decisions about the care of individual patients”*. As EBM is not restricted to medical doctors, the term “evidence-based health care” (EBHC) is used. The process of EBHC starts with formulating an answerable question when faced with a scenario of uncertainty. This is followed by searching for and finding the best available evidence applicable to the problem, critically appraising the evidence for validity, clinical relevance and applicability, interpreting and applying the findings in the clinical setting and evaluating the performance [9, 29].
*Health professions*	All health professionals including doctors, dentists, nurses, occupational therapists, physiotherapists, dieticians, audiologists, mental health professionals, psychologists, counsellors, social workers.

### Participants

We invited EBHC academic programme course convenors/coordinators from training institutions for health professionals across the world by email to participate in the interviews. This purposive selection was informed by considering those publishing on this topic and those participating in an online list-serve on EBHC, administered by the Centre for Evidence-based Medicine, University of Oxford, United Kingdom (www.cebm.net). The aim was to obtain input from a spectrum of programmes covering different health professionals in various countries. We also requested invited participants to nominate key members of their faculty who are involved in EBHC teaching and learning and recommend additional academic programmes, national and international, that could be contacted. We informed participants about the purpose of the interview and asked them to provide written informed consent for participation in the study, for digital recording of the interviews, and for using and disseminating the anonymous information they provided. Participation was voluntary.

### Data collection

Data collection was undertaken using individual semi-structured interviews. An interview guide (Table 3), developed by the investigators through consensus and to align with the objectives, was used to ensure that the same topics of inquiry were covered with each respondent, but at the same time allowing for further exploration of areas of particular interest or concern to respondents, thus creating a rich data set [18]. The interview covered EBHC teaching approaches, and the successes and challenges faced in implementing and evaluating clinically integrated EBHC student programmes, as well as general information on lessons learnt. Interviews lasted an average of 45 minutes and we conducted these in person or by telephone/Skype, at a time convenient for the participant. The interviewer recorded the interview using a digital voice recorder and took additional field notes to ensure full and accurate data capturing. Data saturation was reached by interview 22.

**Table 3:** Interview guide for semi-structured interviews.

Describe your role within the academic programme
What is the definition of EBHC that underpins what you seek to include in your programme?
How would you describe clinically integrated teaching of EBHC?
How is the teaching and learning of EBHC covered in your programme? Give us a short description of the programme and how and when EBHC is covered throughout the course of the programme. What are the objectives? Which EBHC competencies are covered? Please describe the context in which learning take place. Which teaching and learning methods are used? How is EBHC assessed?
What are successes of the programme?
Which challenges have you encountered?
What do you see as the barriers to the integrated teaching of EBHC?
What are the factors that facilitate integrated teaching of EBHC?
What are the lessons you have learnt in teaching EBHC in an integrated manner [if this is being done in the particular programme]

### Data management and analysis

An external company transcribed the interview data for the purpose of analysis. Names of participants did not appear in the transcriptions. The lead researcher (TY) checked all transcriptions by listening to interviews while reading the transcripts, and developed codes and code definitions afterwards. To ensure the transparency of the data coding process, three researchers (TY, SvS and AR) with different backgrounds met to finalise the code book after a preliminary round of independent coding. This code book guided the subsequent coding process [19]. Data were then imported into *Atlas*.*ti*, a software package that facilitates the process of coding qualitative data. The lead researcher coded all transcripts and two co-researchers checked the coding. The researchers then did the data analysis and interpretation using thematic content analysis to identify key emerging themes ultimately relating these to each study objective. This iterative process of aggregation and interpretation was undertaken by the lead researcher and discussed with the rest of the research team. During the discussions, these themes and their relevance to the study objectives were highlighted. Findings were based on individual perceptions while keeping in mind that the teaching and learning context plays a key role. Any lessons learnt were therefore interpreted within these specific contexts.

## Findings

Twenty-four course coordinators from five continents (countries: Australia, Canada, China, Netherlands, New Zealand, Norway, Philippines, Scotland, South Africa, United Kingdom, and the USA) participated in the interviews (Table 4). Interviewees, from mainly medical programmes, were senior academics from a variety of different disciplines, including dentistry, emergency medicine, general practice, internal medicine, nephrology, paediatrics, physiotherapy, and public health. Many had completed postgraduate programmes in clinical epidemiology, had conducted systematic reviews, and linked their initiation into EBHC teaching to attending courses at institutions that championed EBHC, or being supervised for postgraduate studies by international leaders in the field of EBHC. Some had been involved in teaching EBHC to health professionals for more than 10 years, with their own experience in teaching EBHC typically starting at postgraduate level and then extending to undergraduate or graduate medical programme level.

**Table 4:** Characteristics of participants.

Participant number	Gender	Continent	Type of programme
P1	Male	Australia	Medical
P2	Male	Africa	Medical
P3	Male	Australia	Medical
P4	Male	Europe	Medical
P5	Male	Europe	Medical
P6	Male	Australia	Medical
P7	Male	Africa	Medical
P8	Male	Australia	Allied Health
P9	Female	Europe	Medical, Allied and Nursing
P10	Male	Africa	Medical
P11	Female	North America	Medical
P12	Male	North America	Medical
P13	Male	Australia	Medical
P14	Male	Australia	Medical
P15	Male	Europe	Medical
P16	Male	North America	Medical
P17	Female	Asia	Medical
P18	Male	North America	Medical
P19	Male	Europe	Medical
P20	Male	Africa	Medical
P21	Female	Asia	Medical
P22	Male	North America	Medical
P23	Female	Asia	Medical
P24	Male	Africa	Dentistry, Medical

Our findings are presented below in separate sections dealing with the description of the programmes, staff supporting the teaching of EBHC, challenges in integrating EBHC and the critical success factors for successful implementation of clinically integrated teaching and learning of EBHC.

### Programmes structure and process of integrating EBHC

The medical programmes were typically either undergraduate or graduate programmes divided into preclinical and clinical years. Undergraduate medical programmes are 5 to 6 years in duration with students entering directly from school, while graduate medical programmes (GMP) offer 4 years of training to students who have completed an undergraduate bachelor’s degree and may in addition have had some work experience. The size of the student groups taught by participants in our study ranged from 50 students, at a private medical school, to as many as 300 students.

Some EBHC teaching programmes have been running for more than 10 years. Typically in our sample, EBHC teaching started as specific modules coordinated and driven by volunteers, using didactic teaching and with a focus on critical appraisal of research evidence. Over time, opportunities such as curriculum revisions and reform were used to enhance, integrate and formalise EBHC teaching. During these revisions, curriculum assessment, which mapped what was done where, and involvement of those who had been teaching these components, guided joint planning on how EBHC could be integrated. A substantial number of these planning activities were driven by champions *‘planning in (their) backyards’* (P4).

Interviewees explained that the underlying aim with integrated curricula was to have EBHC learning longitudinal, instilled, embedded and part of mainstream. The typical approach for an integrated EBHC curriculum involved laying the foundation in the preclinical years, and linking EBHC learning to specific clinical rotations thereafter. Table 5 provides a summary of the typical content covered, teaching and learning approaches, and assessments used by interviewees.

**Table 5:** Typical integrated EBHC curriculum^#^.

Period of study	EBHC content covered	Teaching and learning approach	Assessment	Selected quotes
Preclinical years	History of and introduction to EBHC principles and practice; Epidemiology principles; Basic statistics; Introduction to library and searching; Approach to critical appraisal	Large group lectures; Small group tutorials; Practical sessions on how to search	Standalone assessments in form of multiple choice questions or short questions	*‘*..*get them to think through problem solving and clinical decision making early*.. *give them basic epidemiology*, *and basic statistics within the whole framework of the doctor / patient relationship*, *and of the doctor and the patient trying to make decisions about their health care and about*, *you know*, *how to achieve that objective’* (P23) *‘You can’t get them to do the critically appraised topics*, *that’s the really integrated part of it if you like*, *unless you’ve got the building blocks in place*, *otherwise they just do it badly*, *so they have to have slowly built up all of the skills so that then they can rapidly do the stuff when they get to the clinical rotations’* (P1)
Clinical years	EBHC linked to specific clinical rotations. Based on a patient seen students phrase a clear question, search for the best evidence, appraise that article using the appropriate appraisal form, interpret findings and consider application to patient. Focus on Diagnosis, Therapy (often main focus), Prognosis, and Risk factor. Communication and implementing evidence in practice.	On the wards / in clinical setting; Small groups doing clinical rotations; Spread out over blocks; Online material to support learning	CATs* submitted as written documents and also presented. Minimum number to successfully complete within clinical years e.g. six over the period. Cover the five core articles types—therapy, harm, diagnosis, prognosis and systematic review. Presented in portfolios which include reflection	*‘Usually where there is a level of uncertainty it’s a good way of leading them into the literature directly from the bedside*. *I hardly ever see a student that doesn’t have a cellphone with internet access…so we would search right then and there to get an answer on a certain topic at risk if it comes up in that*, *on the ward rounds’*. (P10) *‘They have free choice*. *They can pick whatever patient struck them with an interesting patient with an interesting issue*. *Say for example*, *a student was doing a block on surgery*, *at the end of the surgery block; they would have to submit a one-page summary on the standardised form of a question that arose with a patient*. *What their literature search was*, *what their strategy was*, *what paper they found and then a validation and interpretation of the paper they found*. *Then finally*, *how they would explain in their findings in that paper to their patient’* (P16). *‘…*. *so we actually look at every paper and every article and provide our comments about things that the students may not be capturing or particularly good insights that they have developed we try and reinforce those and then we send those back to them*. *So not only do they pass the assignment if it’s appropriate*, *but regardless of whether they pass or don’t pass*, *we trying to provide them some written feedback as well*.*’* (P12)

*Critically Appraised Topic

^**#**^ This table provides a summary of the typical content covered, teaching and learning approaches used, as well as assessments used. This is drawn from content, approaches and assessments named and described by interviewees.

#### Typical EBHC content covered

Generally, the foundation was laid in the preclinical years with topics including the history of EBHC and introduction to EBHC principles and practice, epidemiological principles, basic biostatistics, introduction to library services and searching, and the approach to critical appraisal. During the clinical years, the focus was on asking, finding, appraising, interpreting and considering the use of research evidence related to questions about risk factors, diagnosis, treatment, and prognosis that arose from seeing patients in the clinical rotations–i.e. integrated within clinical rotations and not focusing only on critical appraisal but linking it to interpretation, application and communication of findings. Within the preclinical years, teaching was often included in modules where there was thematic ‘fit’ (e.g. personal and professional development) and, in the clinical rotations, the emphasis was on linking EBHC learning to patients and clinical queries arising within the clinical setting and linking to existing initiatives such as quality improvement projects. Most felt that inserting EBHC into the clinical setting work better with longer clinical rotations i.e. 6 weeks instead of 4 weeks.

#### Teaching and learning approaches

Various approaches were described which had evolved over time. In the preclinical years, much of the teaching took place in large groups and some lecturers employed innovative strategies, such as using videos of clinical scenarios and in class tutorials, to make lectures clinically relevant, more interesting, and interactive. Where resources were available, large group lectures were followed with small group, discipline specific (if a diverse group of students) tutorials where the content covered in the lecture was consolidated. Here, facilitators often chose clinical topics they were comfortable with. In addition, the teaching of EBHC was often linked to either problem based learning or competency based education (e.g. CanMeds). A number of tools (Table 6) [20–22], such as the graphic appraisal tool for epidemiological studies (a tool to guide the appraisal of epidemiological studies), were also used as a way to help students, and faculty, to remember EBHC approaches–‘*something memorable*, *something that’s easy to remember so that it stays with them*.*’* (P6)

**Table 6:** Examples of commonly used tools.

Tool	Short description
AMSTAR	A measurement tool to assess the methodological quality of systematic reviews.[22]
CARL	Clinical Appraisal Research and Lifelong Learning–An approach linking critical appraisal, interpretation of research and application to patients seen in clinical setting
CATS	Critically Appraised Topics–An approach linking critical appraisal, interpretation of research and application to patients seen in clinical setting.
GATE	Graphic Appraisal Tool for Epidemiological studies - A tool to guide the appraisal of epidemiological studies. [20]
PEARLS	Presentations of Evidence Abstracted from the Research Literature to Solve the real patient problems—15-minute presentations given by students addressing a focused clinical question raised by their contact with a real patient during a recent clinical attachment. [21]
PICO	Approach to phrase a clear question: P- Participants I–Intervention/Issue C- Comparison/Context O- Outcome

Participants highlighted the importance of teaching being relevant to students explaining that students start their training motivated to become healthcare professionals and, especially in the preclinical years, need to see the relevance of EBHC to being a healthcare professional. This increases their interest and facilitates learning. Outlining the clinical context and how EBHC fits into healthcare decision making were therefore identified as being important. This was, however, not easy to achieve during the preclinical years. Some lecturers found that using topical examples, technology (e.g. using videos of clinical scenarios) or both was helpful in bringing the clinical setting into the lecture room.

In the clinical years, critically appraised topics (CATs), where students apply the various steps of EBHC to a patient seen in the clinical setting, were typically done in small groups with a tutor linked to the group. Students started by identifying topics based on patients they had seen, then phrased a clear question, searched for evidence, appraised and interpreted the evidence and considered issues related to application of the evidence to the patient. The approaches used, in both preclinical and clinical phases, however were often dependent on the size of the class, available resources and available teaching opportunities. Despite recognition of the potential value of using technology such as use of mobile phones, tablets, etc., this was not a key approach described by participants.

#### Assessment

Both formative and summative assessment was used. Typically, standalone assessments were used in the preclinical years and more integrated assessments in the clinical years. In the preclinical years, assessment examples included short questions, online quizzes and multiple choice questions (MCQ) while in the clinical years the submission, and in some instances individual or group presentations, of CATs were a curriculum requirement. During the presentation of CATs, students presented to their peers and lecturers, and peer assessment of these was used at some institutions. Individual as well as group feedback was given to highlight key issues that students were struggling with, mostly related to critical appraisal. As these were completed over various clinical rotations, some institutions included the CATs, the feedback and a reflective report by the students within a longitudinal portfolio.

### Who is supporting the teaching of EBHC?

Interviewees had dedicated roles with regard to EBHC teaching, which were linked to new programmes or involved curriculum reform in an established curriculum. Their roles included teaching, curriculum review, evaluation, setting and grading assessments, and working with others to ensure continuity in the programme. For established clinically integrated programmes, the interviewees perceived their main role to be ensuring quality assurance. EBHC teaching was however just one component of their general academic or clinical responsibilities. *‘I mean it’s only a small part of what I do’* (P6).

**Fig 1:**
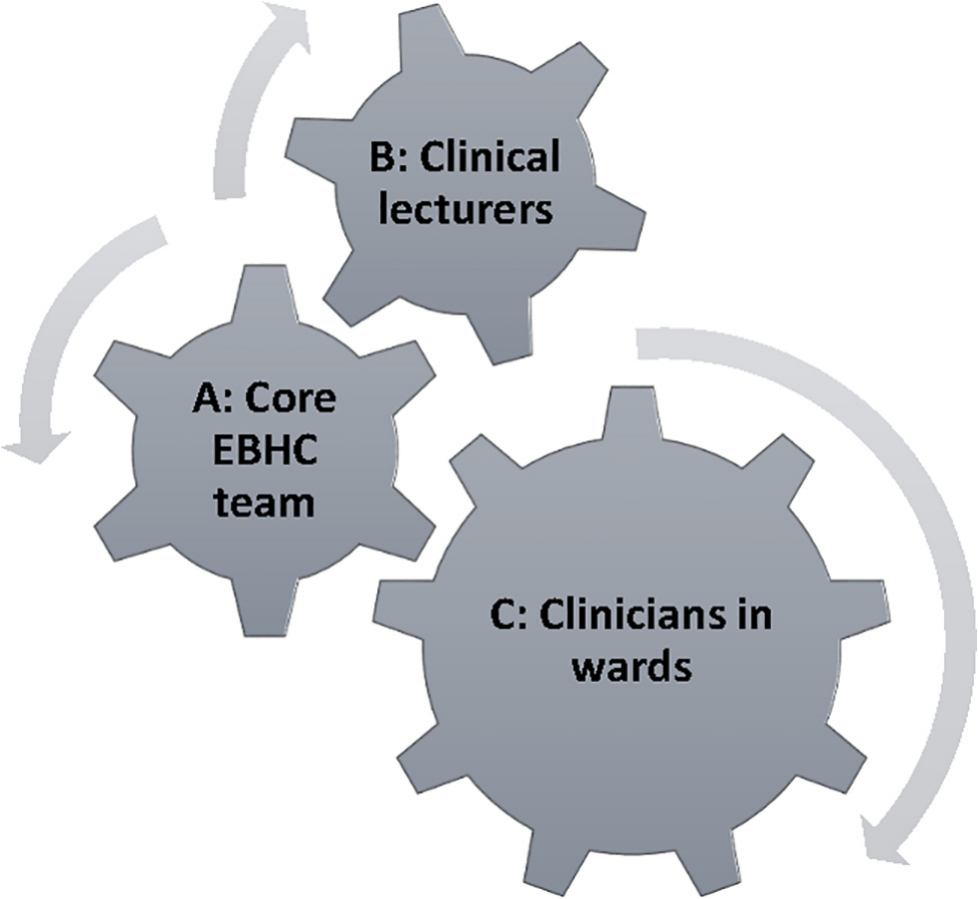
Staff involved in facilitating learning of EBHC.

Staff involved in facilitating EBHC learning typically fell into three groups–the core EBHC team, clinical lecturers and clinicians working in the clinical setting (Fig 1). The number and extent of people involved depended on how integrated the EBHC programme was, the size of classes and the availability of funding to support teaching. Core EBHC team members were few in number and usually trained in EBHC, experienced in conducting systematic reviews and other research, committed, enthusiastic, and comfortable with uncertainty. They were supported, in some cases, by tutors or teaching assistants (e.g. PhD epidemiology students), librarians and, rarely, by administrators. This team typically initiated EBHC teaching and learning and continued to drive the process.

The clinical lecturers were often academics from various disciplines, who were teaching students. They usually had postgraduate qualifications in clinical epidemiology or epidemiology and an interest in EBHC, clinical epidemiology or public health. Some were medical or non-medical researchers working in a clinical setting. Their involvement usually started through either participating in continuing professional development or postgraduate training in clinical epidemiology or EBHC.

Clinicians working on the wards play a critical role in facilitating the learning of EBHC as students look up to them as role models. The core EBHC team and clinical lecturers in some cases teamed up with the clinicians in the wards and, through their complementary backgrounds and roles, facilitated linkage between EBHC and clinical expertise.

### What challenges did they experience in integrating EBHC in clinical settings?

Despite dedicated attempts to integrate EBHC learning in clinical settings, not all programmes were truly integrated. Some were running EBHC as separate courses within the programme e.g. 8 weeks of EBHC with lectures and tutorials where all the activities are classroom based. By far the most common challenges related to lack of space in the clinical setting, EBHC misconceptions, resistance of staff and lack of confidence of tutors, time, and negative role modelling. Issues that were also noted in some instances were student commitment and quality assurance.

#### Lack of ‘space’ in the clinical setting

*‘…treading on someone else’s curriculum real estate*.’ (P11); *‘*..*my content*, *my space’; ‘Well the politics of treading on other people’s territory’* (P1) Curricula were typically full with each discipline jealously guarding the inclusion of their content in the curriculum. In addition, competition for teaching time and space, while dealing with the patient responsibilities in the clinical setting, was a key challenge faced by many.

#### Misconception or lack of knowledge of EBHC

Knowledge and attitudes towards EBHC varied amongst faculty members at the different institutions. Misconceptions that EBHC was only based on evidence from randomised controlled trials, or only focused on the evidence and critical appraisal and ignored patient values and clinical experience, hampered the facilitation of learning of EBHC in the clinical setting.

*‘The faculty are not to the standard of the students*, *and so when the students get into that clinical environment*, *they basically are not supported by faculty*, *except in a few departments*, *and a few individuals*.*’* (P20)

‘*The other major challenge I mentioned is that as long as we’re not able to teach the teachers*, *or make sure the teachers integrate this in clinical teaching*, *then our efforts are*, *if not futile*, *they’re not very effective*.*’* (P19)

*‘Many of the old guys (are) traditional and very difficult to move especially clinicians and it is clear that they don’t sort of keep up to date in this field’*. (P24)

*‘*..*the blind leading the blind*, *because you know a lot of clinical teachers are not well trained in evidence based medicine*, *critical appraisals*, *so you kind of have the students in a way*, *sometimes know more theoretically than their clinical teachers*.*’* (P6)

#### Resistance among clinical staff and negative role modelling

As a consequence of workload, limited knowledge (and also feeling threatened) or misconceptions about EBHC, some staff in the clinical environment did not see the need, value or relevance of EBHC teaching in the clinical setting. As a consequence, they blocked or inhibited attempts at clinical integration. As students do what they see, and pick up that clinicians do not practice EBHC, the impact and influence of negative role modelling in the clinical setting may be far-reaching.

*‘So what we fear is that we teach them*, *they learn quite a lot in the classroom*, *they can even practice it on paper and pencil exercises*, *but when it actually comes to the clinical time*, *it tends to be undone by the negative role model of people who don’t do it*, *whom they revere as teachers*, *clinical teachers’*. (P3)

*‘So the resistance was–we don’t believe you*, *we think it’s worthless…’* (P5)

*‘However*, *some of the older consultants I think experience it really as a threat and especially if the students start knowing more about that than they do and then they sometimes feel threatened by and often make it as unimportant or ignore it’* (P10)

*‘They don’t do it*, *they don’t know how to do*, *they don’t see any value in it and so the students pick that up very quickly*. *They see that they*, *you know*, *we do it this way because we’ve always done it this way*, *because I know its best’* (P3)

#### Staff lack of confidence to facilitate learning of EBHC

Lack of confidence impacted on integration in the clinical setting as staff in clinical departments were not comfortable to teach EBHC, to encourage critical enquiry, to facilitate EBHC learning or administer related assessments.

*‘…what’s compounded it is that none of these skills are being demanded of them by the clinicians in the ward*, *and so basically we deliver a curriculum that doesn’t–that does demand in terms of assessment and marks*, *but is not carried forward into the clinical years to any real degree*, *and the student skills are … I think they appreciate the concepts*, *but I don’t think they’re actively applying it*, *now in the day to day work*.*’* (P20)

#### Lack of time

Lack of time for teaching EBHC, setting and grading assessments, providing feedback to students on their assessment performance, or for faculty development initiatives, etc. was identified as a further challenge. Dedicated time was seen as important to develop and standardize teaching resources and material, assessments, and marking schedules. Furthermore, on an ongoing basis, tutors need training, guidance and oversight, teaching material needs refinement, and teaching sessions require preparation, reflection, online engagement (in some cases), engagement with clinical staff, marking of assessments and feedback to students. All these are however only one part of academic life and participants highlighted the challenge of balancing academic life–teaching, research, grant writing, supervision of students and other priorities. Those involved in the teaching of EBHC enjoyed doing it and often did it out of a sense of passion but burnout was raised as a real concern.

*‘So it took a long time for each session when I first developed it … I have to read the paper and design the work sheet*. *I then have to mark the worksheets which takes me two hours for 15 worksheets and then*, *I have to you know develop slides based on the problems with the worksheets*.*’* (P11)

*‘*.. *it was very important for us that there be a substantive feedback component to our evaluation*.*… The challenge with that is it is labor intensive and it is time intensive*. *That’s a commitment it’s an investment and you either have to have a lot of graders if you have a lot of students or you have to have substantial time commitment from the medical school*.*’* (P12)

*‘… and then on an ongoing basis I would say that the whole thing is probably about a half day*, *not including the face time of teaching*. *You know tweaking the slides*, *putting out the materials*, *marking the worksheets and then going and doing the sessions and some of the sessions you know I have to update them*.*’* (P11)

#### Student commitment

Students want to practice medicine and don’t always see the relevance of EBHC within the curriculum. Where EBHC is seen as an add on and not as part of the core curriculum, their level of commitment in engaging in and preparing for EBHC activities varies. Those doing the GMP were found to appreciate the relevance of EBHC earlier than their undergraduate counterparts.

*‘I think that’s the biggest challenge in undergraduate teaching in second year is that they don’t have a context for it getting to understand the importance of it*.*’* (P8)

*‘… teaching EBM to the undergraduate students is very*, *very difficult*, *because it’s very abstract for them*, *…*. *it’s very difficult for them to understand*, *…*. *what I do is in many instances I take a step backwards*, *give them a clinical scenario and try to explain to them in a practical way what they understand about it*..*’* (P2)

*‘The trouble is that students don’t terribly like it*, *you know*, *they want to get on and cure people*. *Learn how to make diagnoses and all that stuff and the stuff about looking up things on a computer and worry about what an odds ratio is and interpreting confidence intervals*, *it all seems rather remote from direct patient care’*. (P3)

#### Quality assurance

Various levels of staff across different disciplines were involved in the teaching of EBHC. It was felt that standardisation and quality assurance of the teaching and learning of EBHC are needed to ensure that consistent messages are provided to students.

*‘think it’s*, *you need to believe in EBM and I think you need to practice that and you have to believe in that*, *you know it’s like a religion*, *you have to think of it and you have to practice it*, … *the core group which teaches the EBM*, *you don’t have a capacity to go into every department and do this and it would have been ideal that every clinical departments have somebody who is championing EBM and teach them in their blocks*, *but that didn’t happen…*.’ (P2)

### What are the critical success factors in successful implementation of clinically integrated teaching of EBHC? (Table 7)

**Fig 2:**
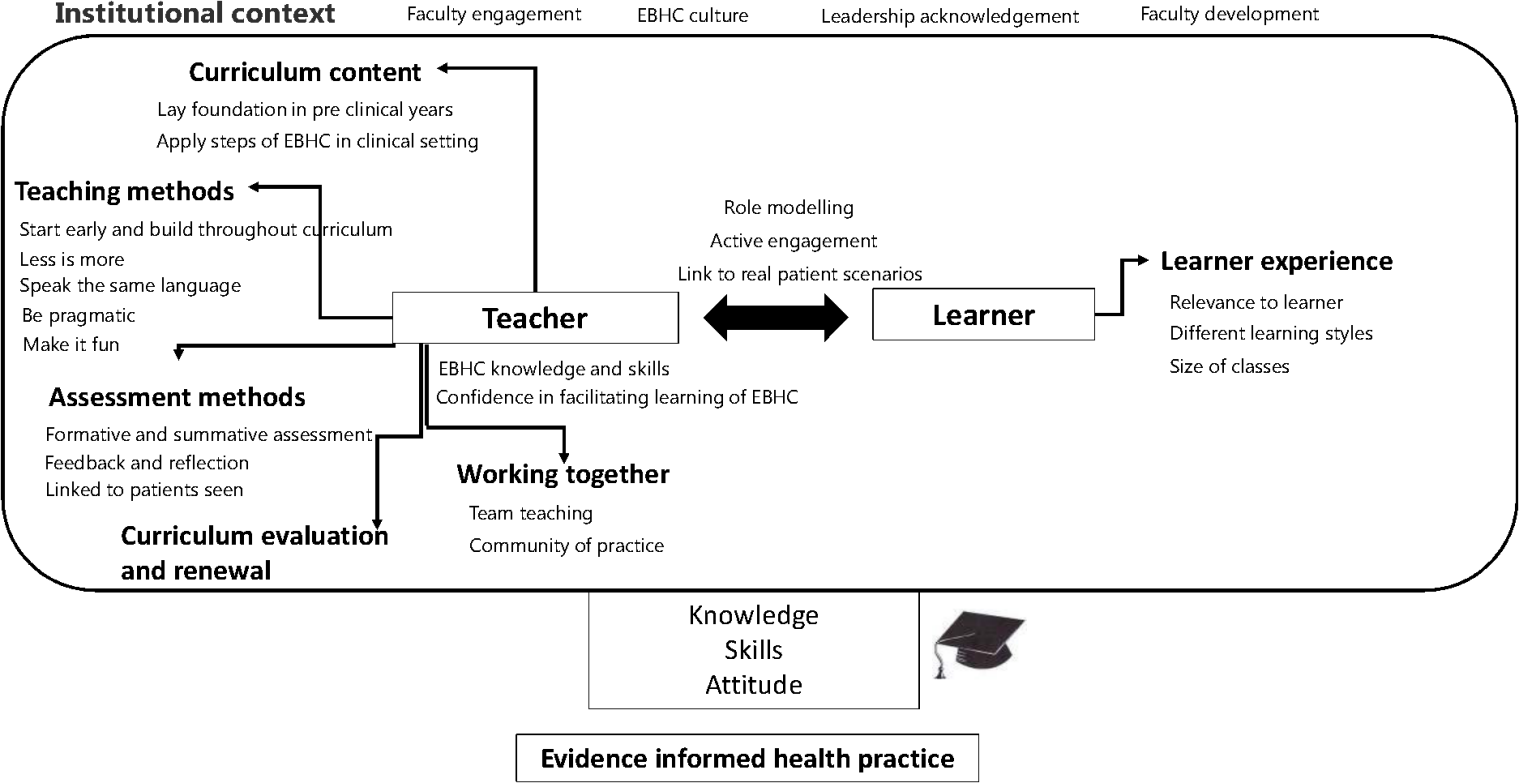
Concept map with key issues for integrated teaching and learning of EBHC.

Those who successfully implemented clinically integrated teaching of EBHC shared what they regarded as key success factors. By far the most common factors were being pragmatic, and patient, starting early in the curriculum and building from there, leadership acknowledgment and the right teachers and role models. Issues that were also noted in some instances were evaluation and curriculum renewal, having a community of practice, and a culture of EBHC. We include some illustrative quotes in the text below. Fig 2 brings together the various findings in the form of an overarching concept map.

**Table 7:** Critical success factors in successful implementation of clinically integrated teaching of EBHC.

Be pragmatic
Patience and persistence
Start early, build from there with relevant examples using a variety of delivery and assessment methods
Need the ‘right’ teachers
Role modelling
Evaluating teaching and curriculum renewal
Leadership acknowledgment, faculty engagement and institutional culture of EBHC

#### Be pragmatic

There is ‘no one size which fits all’ when it comes to clinical integration of EBHC. Taking the contextual factors into consideration, programme coordinators identified opportunities, such as when the curriculum was being revised, and used these to work with others to integrate EBHC teaching and learning. Linking and integrating EBHC with existing teaching was also considered important e.g. as part of quality improvement projects which was a good way of showing how EBHC links to existing healthcare processes and practice. Furthermore, not all clinicians are interested in EBHC and EBHC champions thus chose to start work with ‘early adopters.’

*‘*..*you have what you have and you have to work within that … there’s no one curriculum you can hand people*, *because of the structures of most medical curriculum’s*, *you’ve got to sort of bend it into shape to make the principle’s fit’* (P1).

#### Patience and persistence

Integrating EBHC took time. Participants described the time it took to understand what was happening in the whole curriculum, what the needs were, what the programmes covered, to find space where EBHC could fit in, to encourage or convince others to link EBHC learning to their rotations and then work with them to integrate EBHC and build capacity and confidence in facilitating the learning of EBHC. This process of change management required patience and persistence.

*‘There’s no quick fix…*.*it’s a never-ending process*, *so we gradually put those building blocks in place over several years*, *it was getting better and better*.*’* (P1)

*‘Then you just have to be patient*, *patience is very important in–if you’re about to introduce something new*. *When I say patient*, *you cannot change everything in one flick of a finger*. *You*, *you know*, *change is actually very slow*, *but if you’re patient you know*, *it will come*. *So*, *that’s something that we have learned*, *and we’ve been doing it for 20 years*, *and now we don’t even have to do much*, *people just … people who have already learned about this*, *are the ones doing the talking*, *and incorporating the activities*, *wherever they are*…*’* (P21)

‘..*don’t give up or compromise on the larger goal and so what I mean by that is–in my opinion evidence based medicine—to be relevant as part of a curriculum absolutely has to be integrated*… *don’t compromise that goal because it will be easier to compromise it because integrating is hard*, *but I think it would be to the detriment of your curriculum*.*’* (P12)

*‘…take small steps at a time to build up to that stage*. *I think that’s–to all of us that’s a very desirable end point*, *but that may take some time to be achieved’*. (P18)

#### Start early, build from there with relevant examples using a variety of delivery and assessment methods

The need to start EBHC teaching early in the pre-clinical years to lay the foundation and then to repeat and scaffold EBHC learning throughout the programme to ensure continuity came up consistently. In doing so, a few key approaches were highlighted. Interviewees mentioned linking teaching and learning to real patient scenarios to engage students and also to let them appreciate the relevance. They recommended that one must avoid linking it to ‘boring’ subjects, and should consider the different learning styles of students, and their diversity. A variety of delivery methods can be used, where one should try not to cover too much (‘*less is more’*), and also make it fun. Furthermore, interviewees highlighted involving clinicians in the ward as part of the teaching team, speaking the ‘same language’, and including assessment (as it drives learning). Providing feedback to students on their performance was also highlighted.

*‘That student could consider using evidence-based medicine and looking articles up as much as they would consider listening to a patient’s chest with a stethoscope or looking at their eyes with an ophthalmoscope*.*’* (P16)

*‘So*, *I would say you have to make the*, *the key learning point*, *make the learning experience*, *spokes on the learning experience to make (it) fun*, *engaging*, *break up your sessions from*, *from larger groups to smaller groups*, *wherever possible*. *Engage the students to think for themselves*.*’* (P15)

*‘*..*in a variety of formats so we’ll do both face to face as well as online teaching like I mentioned before role play and integrating it with the existence PBL activities and clinical bedside teaching and activities as well so it’s not just I guess one model of delivering the course …*.*a multifaceted approach which the students have enjoyed*.*’* (P13)

*‘…*.*they’re team taught…which brings credibility to the subject matter’* (P11)

*‘I think the other successors of the program were the branding…*.*So branding and recognition for the students has been pretty key in their understanding that this is a skill that is woven throughout and reinforced at increasing levels of sophistication*.*’* (P11)

#### The ‘right’ teachers

Interviewees felt that integrating EBHC relied on an enthusiastic and committed critical mass of teachers with requisite knowledge, attitudes and skills. To achieve this, there was a need for continuous sensitisation of staff to the importance of EBHC, provision of guidance and support in how best to integrate EBHC into a curriculum, and recognition or reward of EBHC champions. Faculty development initiatives were often targeted at the new generation of health professionals and focussed on improving understanding of EBHC and how to teach EBHC. It included dedicated capacity building sessions, sharing useful resources, and supported learning while part of the teaching team. Such initiatives enhanced knowledge, confidence, and attitudes towards EBHC and often served as an opportunity to identify potential tutors/teachers. Those working at institutions which had established postgraduate masters or doctoral programmes in clinical epidemiology described having achieved a critical mass of graduates working within clinical departments who were leading the teaching of EBHC in undergraduate programmes.

*‘We had a PhD programme for clinical epidemiology seniors*, *and most of these persons were involved afterwards*, *after they complete this programme…*.*And then you can keep them attached to your plans*, *and then these are people who can send a message in the hospital that they fare better*.*’* (P5)

*‘*.. *I built up my own internal tutors and including tutors across the other programs*, *so I would get people in surgery interested in learning about evidence based medicine*, *would get a place in the workshop and that’s a long-term program and that’s building up the capacity that you have*.*’* (P1)

*‘…how best we can teach*, *how can we teach teachers how to teach EBM in an attractive way*, *to our students’* (P2)

*‘We’ve been embracing*, *if anyone shows an interest*, *they’re welcome to join the team*.*’* (P20)

‘*Stop devoting time on teaching the old ones*, *I’m going to devote my time particularly to scouting highly talented young ones…’* (P5)

#### Role modelling

Seeing others, in particular their teachers, clinicians (especially those who they respect) and their peers, practicing EBHC was the most powerful tool to facilitate learning of EBHC by students.

**‘** *The most important way of teaching students evidence based medicine is by example…*. *It is not what we say but what they see we’re doing*…*’* (P10)

*‘If you don’t read they can see that you are reading*, *if you don’t look and do research… that they can see that you also do not know*. *I think the biggest illusion and the biggest problem with our training nowadays is we want students to believe that we know everything and that’s our biggest shortcoming*. *The students need to know that their professors and their doctors or their teachers do not know everything and constantly seek to find out what is going on and go and read up and know where to get the information*, *I think that’s the … probably the biggest place where you can teach your students to learn evidence based medicine is to acknowledge you don’t know*, *you sit with a problem*, *with a patient*, *I don’t know what it is*, *but let’s find out*.*’* (P10)

#### Evaluating teaching and curriculum renewal

Curriculum review played a key role in informing the integration process. It provided opportunity to map what was being taught where and identified opportunities for inclusion of EBHC. Ongoing evaluation of what works, what could be done differently, and being aware of new developments in the field, were key to keeping the EBHC curriculum up to date and relevant. Also highlighted was the need to ‘move with the times’–keeping track of what is new, what content could be covered and modern approaches to teaching e.g. use of e-learning and social media as teaching tools.

**‘** *I continue to modify and try to tweak it*.*’* (P6)

*‘So the first thing I did is*, *the lead of the thread*, *was to find out what other related teaching was appearing in all of the other disciplines*, *so I found out that some basics sciences were teaching some critical appraisal and statistics in the early years of the course*, *that the librarians were teaching a bit about searching but the surgeons in the fifth year had a critical appraisal afternoon within their program*, *etc*., *so I mapped all of that out and then got the people together who were doing this and said*, *OK*, *if we’re going to have an integrated course*, *a thread that goes across all the years*, *what are the upper laps*, *what are the connections*, *what are the missing bits across this thread*.*’* (P1)

#### Leadership acknowledgemnt, faculty engagement and culture of EBHC

EBHC is a philosophy **–‘** *it’s about learning*, *we are all learners we can learn everything*, *we don’t know everything’* (P21). This institutional culture of critical enquiry was considered an important contextual element to foster EBHC learning. Furthermore, the degree of recognition by faculty leadership, and the resources provided to support EBHC teaching initiatives were regarded as important. These ranged from some interviewees having little to no dedicated resources and teaching of EBHC being seen as an expected element of their academic work, to having dedicated funding for both academic and administrative posts to support EBHC teaching. Formal acknowledgment and endorsement of the EBHC curriculum by senior leaders and management created enabling environments for the implementation of the teaching and learning. This translated into support, which could be verbal, monetary or related to provision of resources, and importantly, also influenced how students see EBHC. Engagement of senior faculty, from all disciplines, further supported implementation.

*‘*..*we have very good I mean superb support from our medical school leadership they understand that evidence based practice is something that our graduating students must be familiar with and they support that and because of that support that comes through to the students over time as well and they understand look*, *I have to be familiar with these principles*, *this curriculum is going to help me to be familiar with these principles and this is something that is going to be relevant to my clinical practice*, *this is not just make work for the student*.*’* (P12)

*‘In terms of getting other people engaged*, *so you can have all the resources you like*, *but unless you can also use that resource to lever engagement in the other disciplines*, *then you don’t encroach on the hidden curriculum*.*’* (P1)

**‘** *If you have leadership support …*.*everything else follows from that’* (P12)

*‘Other than saying it’s*, *it may be … it’s humbling to say that you don’t’ know and I think certain disciplines there’s people that’s … just afraid to say well I really don’t know and I think that’s the bottom line of evidence based is just acknowledge that you don’t know’* (P10).

#### Community of practice

Working and networking with like-minded people involved in the teaching and learning of EBHC–having an opportunity to reflect and engage regularly on these issues–within a community of practice not only as a support network but also as a group to further the teaching and learning of EBHC were raised by most participants. This included engaging on aspects such as how best to facilitate EBHC learning, standardise teaching resources, validate assessment procedures and tools, and building consensus around what the EBHC curriculum should include.

‘.. *a national or international network of people … and some kind of forum where you could get together and talk about issues particular to EBHC*, *curriculum development’* (P11)

## Discussion

Worldwide, academic institutions are including, or considering the inclusion of, teaching and learning of EBHC in health professions curricula [9]. The recent paper by Greenhalgh and colleagues [23] draws attention to clinical training playing a key role in supporting the delivery of ‘real EBHC’. However, little is known about how to implement clinically integrated EBHC teaching and learning. This study reports on lessons learnt from those who have successfully implemented, or who have attempted and failed to implement, clinically integrated EBHC teaching and learning [10, 12]. It describes approaches used, successes and challenges faced, and lessons learnt in teaching and learning of EBHC in an integrated manner in order to better inform future implementation strategies. Participants were from various countries and the themes, both challenges and successes, were consistent across countries.

Implementation of clinically integrated teaching and learning of EBHC takes much time and many programmes did not have full integration of EBHC learning in all clinical rotations. Typically, learning started in pre-clinical years through the use of real clinical scenarios and subsequently was consolidated with application to real patient settings and assessment within the clinical years. The EBHC curriculum content needs to cover the full spectrum of EBHC and not be focused on critical appraisal only. On-going curriculum revision and renewal are needed before integration can become ‘business as usual’. Medical curricula are however typically organised around disciplines and this is often a barrier to integrating cross-cutting issues such as EBHC. A holistic approach to curriculum renewal, recognising that this might require a change management process, is needed. Critical success factors were adopting a pragmatic approach and being ready to use opportunities for engagement and for fitting EBHC learning within the curriculum, patience, and a critical mass of the right teachers who have EBHC knowledge, attitudes and skills and are confident in facilitating the learning. Role modelling within the clinical setting emerged as a critical facilitator. The institutional context has an important influence on what is possible [24]. Faculty buy-in, endorsement by institutional leaders and having an EBHC culture, together with a community of practice, create an enabling environment. By far the most common challenges were lack of space in the clinical setting, EBHC misconceptions, resistance of staff and lack of confidence of tutors, time, and negative role modelling.

Our study findings are consistent with those of similar studies. A survey conducted by Oude Rengerink and colleagues [14] on barriers and facilitators for teaching EBM in clinical practice (as part of continuing education) in various European countries found lack of teaching time and lack of EBHC requirements in curricula as key barriers, and train the trainer initiatives and access to relevant databases as the key facilitators. Dans [4] highlighted the lack of role models in the clinical setting as an important limitation which could be overcome over time through postgraduate EBHC programmes and train the trainer initiatives. Through interviews with undergraduate medical students, Ilic [25, 26] found that demonstrating applicability to clinical disciplines and mentorship are key facilitators while lack of application by senior clinicians was a main barrier. Issues which emerged however are not always unique to EBHC but resonate with teaching and learning in general especially related to other cross cutting themes such as ethics [27] and inter professional education [28].

Our study focused on the experience in EBHC teaching and learning at pre-service level, from the perspective of academic programme course convenors/coordinators from training institutions from across the world. Even though contextual factors, such as the culture of EBHC, change over time and barriers from 15 years ago might have reduced over time, this qualitative study sheds further light on both potential barriers and facilitators to the implementation of clinically integrated teaching and learning of EBHC. It adds to the knowledge base by sharing experiences and lessons learnt in how to implement clinically integrated EBHC teaching and learning, an issue many are, and should be, grappling with. EBHC provides an approach towards enhanced health care and thus fits within the calls for a shift in health professions education [2] and the shift from memorization of facts to *“critical reasoning that can guide the capacity to search*, *analyse*, *assess and synthesise information for decision-making”*.

Strengths of our study include the international scope of the participants who are linked to institutions in various regions, and the trans-disciplinary nature of the research team with postgraduate academic backgrounds in medicine, nursing, evidence-based health care, public health and higher education. The lead researcher, with a background in public health and EBHC, conducted all the interviews and led the coding after the lead researcher and two other researchers, with different backgrounds, discussed and finalised the code book. Members of the research team are involved with teaching and learning of EBHC at their local institution and therefore have a special interest in this research topic. While a potential limitation of our study is that most participants were involved with medical programmes, the experiences and lessons learnt from medical settings seemed to resonate with those reported within other programmes. We recognise that there may be differences between countries but we were limited by what was covered in the interviews and a deeper understanding of this requires a detailed study at institutional and national level.

## Conclusions

Clinically integrated teaching and learning strategies are the best strategies to build EBHC knowledge, skills and attitudes of new health professionals. Implementing such a curriculum requires institutional support, a critical mass of the right teachers and role models in the clinical setting, and most of all patience, persistence and pragmatism.

## Supporting Information

S1 File Consolidated criteria for reporting qualitative research (COREQ) checklist. (DOCX)
